# Dr. Stokes, on Erysipelas

**Published:** 1800-10

**Authors:** Jonathan Stokes

**Affiliations:** Chesterfield


### To Dr. BRADLEY.

Dear Sir,

We are obliged to Dr. Wells * for communicating to the public, the obſervations which have induced him to adopt the opinion of thoſe, who hold that Eryſipelas of the Face is a contagious diſeaſe. This opinion was never advanced by the late Dr. Cullen,† though noticed by him. "This diſeaſe," ſays the Profeſſor, "is not commonly contagious; but as the diſeaſe may ariſe from an acrid matter externally applied,(1) ſo it is poſſible that the diſeaſe may ſometimes be communicated from one perſon to another".(2)‡ I will now give you his commentary on this text, as I wrote it down, I believe, at the ſame time that Dr. W. took his notes.

(1) "The ſting of a bee or waſp, I have ſeen produce all the effects of eryſipelas of the face; and it is probable, that acrimony may be communicated from one perſon to another."

(2) "An hoſpital phyſician, of ſufficiently juſt information, ſays, that they have had inſtances of the contagious nature of the diſeaſe, ſeveral perſons being affected in the ſame room or ward. That it has been ſo communicated is a fact; but I have a hundred negative inſtances, in which only one in a family has been affected, though there was a free communication between the patient and the other branches of the family."

I obſerve, that in my copy of the Profeſſor's text book, I have drawn a line through the above paragraph; but whether by the Profeſſor's direction or not, I cannot call to mind.

Now, what are we to think of Cullen from the text and the commentary? He admits the fact communicated by the hoſpital phyſician, but alleges, that he has a hundred negative inſtances to prove the contrary. Has not every one ſeen a hundred inſtances of typhus, in which only one in a family has had the diſeaſe? But ſhould we be juſtified in aſſerting, that ſuch a diſeaſe was not contagious? Dr. Wells's concluſion is, that the Profeſſor was "either unconvinced of its truth, or was unwilling to acknowledge it;" but, if the Doctor had continued to peruſe the whole of the chapter, he would have found the following paſſage; "We have hitherto conſidered eryſipelas as, in a great meaſure, of a phlegmonic nature; and

---

* Med. and Chirurg. Trans. V. ii. p. 213.

† Ib. p. 214.

‡ Firſt Lines, 1779, V, ii, p. 8. (not 228, as in Med. and Chirurg. Tranſ. V. ii. p. 222.)

agreeably to that opinion, we have proposed our method of cure. But it is probable, *that an erysipelas is sometimes attended with, or is a symptom of, a putrid fever*;(3) and in such cases, the evacuations proposed above may be improper, and the use of the Peruvian bark may be necessary;(4) but I cannot be explicit on this subject, as such putrid cases have *not* (5) come under my observation."(6) p. 14.

My commentary on the above paragraph is as follows:—

(3) "The carbuncles in the plague are a kind of erysipelas: If a symptom of putridity, it is probable that there is always a mixture of putrescency in erysipelas."

(4) "Or indeed our principal remedy; though, in true inflammation, highly improper."

(5) "Hardly."

(6) "In some cases the bark has been employed, not only with safety, but with apparent advantage; but such cases are of rare occurrence: It has been found necessary in the London hospitals. In great cities there are more sources of putrefaction; and in such cities, hospitals are chiefly found; whence the greater frequency of such cases in such situations."

Dr. Wells's cases, therefore, confirm the opinion of that excellent teacher, whom, however I may differ from him on the theory of fever, I shall venture to call the best writer on medicine of the eighteenth century, that "it is probable that erysipelas is sometimes attended with, or is a symptom of, putrid fever." Dr. W's two first cases, which occurred in May 1796, appear to me to have been instances of typhus erysipelatosa, the erysipelas typhoides of Sauvages.* Of the nature of those cases of erysipelas of the face, which occurred in August and December, I do not presume to judge, my experience, like that of Cullen's, having been hitherto of the negative kind. Such of your Correspondents as keep notes of their cases, may, perhaps, assist us in determining their nature. On the 7th of October, I had a case of erysipelas of the face, and another on the 9th of January, 1797. Typhus was the prevailing disease of September. I do not remember ever to have lost a patient in erysipelas of the face. Whether this has been the result of accident, or to my having seldom, if ever, employed venæsection, confining myself to natron vitriolatum, hydrargyrum, and antimonium tartarisatum, with the occasional use of cinchonæ, is uncertain. What is the nature of erysipelas of the face in the fen countries? Are not the diseases of London, in some measure, still affected by the low grounds of Essex?

I take

* Nosol. i. 450.

I take the liberty of fubmitting to the confideration of Dr. Wells, whether, if the Eryfipelas of the Face be at any time a really contagious difeafe, we may not expect to find, as in moft contagious maladies, perfons of all ages in the fame family affected with it. I may add too, that "perfons who have once laboured under this difeafe," as Cullen obferves, "are liable to returns of it." Now, in contagious difeafes, the reverfe has been obferved. Thofe who have had the fmall-pox are fo feldom liable to be affected a fecond time, that every one confiders himfelf as fecure from the difeafe. The fame perfon very rarely has the chin-cough; and thofe who have gone through typhus, are certainly lefs liable to receive the infection.

In the volume which contains Dr. Wells's Obfervations, is a reflection which, as a botanift, I cannot allow to pafs without animadverfion:* "Bitter medicines," fays Dr. Fordyce, whofe ingenious obfervations on our art merit the thanks of all his brethren, "fuch as the bark of the cinchonæ, feveral fpecies of the artemifia of Linnæus, who, it may be obferved, *has conftantly endeavoured to miflead ftudents in medicine*, by taking the name of the genus from the fpecies which has the leaft effect, or a diffimilar effect from the greateft number of the fpecies in it, &c.†" Linnæus was not without his weakneffes; but nothing could be farther from his intention than to miflead the medical ftudent, as is evident from his Materia Medica, Amoenitates Academicae, Flora Lapponica, Flora Suecica, &c. &c. Artemifia, Abfinthium, and Abrotanum, all Greek names, diftinguifhed as many genera in the fyftem of Tournefort; Linnæus very properly reduced them to one. "Nomina generica poetica, deorum ficta, regum confecrata, et promotorum botanices promerita retineo," is one of his canons in his *Philofophia Botanica*.‡

To Artemifia he gave the preference, in honour, as he fuppofed, of the Queen of Cafia,§ though it more probably received its appellation from the fame origin as Artemis, the Greek name of Diana, that is, from *Artemees*, fafe; both the herb and the goddefs being believed to preferve the mother in child-birth. Pliny fays, "Ab Artemide Ilithyia, quoniam privatim medeatur foeminarum malis."‖

*Chefterfield, Aug. 21.* JONATHAN STOKES.

* Dalech, 1599.

† Med. and Chir. Tranf. V. ii. p. 325.

§ Ib. p. 171.

‖ Hift. Nat. xxv. c. 7, p. 636.

